# Experiencing Cerebrovascular Diseases like Stroke and Fear of Falling: Longitudinal Results from the Survey of Health, Ageing and Retirement in Europe

**DOI:** 10.3390/geriatrics9050133

**Published:** 2024-10-11

**Authors:** Agon Tahiraj, Hans-Helmut König, André Hajek

**Affiliations:** 1 Department of Neurology, Asklepios Hospital Wandsbek, Alphonsstraße 14, 22043 Hamburg, Germany; 2 Department of Health Economics and Health Services Research, Hamburg Center for Health Economics, University Medical Center Hamburg-Eppendorf, Martinistr. 52, 20246 Hamburg, Germany; h.koenig@uke.de (H.-H.K.); a.hajek@uke.de (A.H.)

**Keywords:** stroke, fear of falling, cerebrovascular disorders, SHARE

## Abstract

**Objective:** The aim of this study was to clarify the link between experiencing cerebrovascular diseases (strokes as an explicit example) and fear of falling (FOF) among middle-aged and older adults in Europe. **Methods:** Longitudinal data were used from wave 5 to wave 7 of the representative Survey of Health, Ageing and Retirement in Europe (SHARE). Self-reported tools were used to quantify the key variables. Fear of falling was similarly assessed using a dichotomous yes or no question, “For the past six months at least, have you been bothered by any of the health conditions on this card”, with fear of falling being one of the options. It was adjusted for various sociodemographic and health-related factors. In particular, to account for unobserved heterogeneity, conditional fixed effect regressions (FE) were used. Accordingly, change in an individual’s FOF status over the included waves was analysed and correlated with the reported change of all the included time-varying independent variables within the same individual, including experiencing stroke or other cerebrovascular diseases. The final analytical sample equalled *n* = 22.071 observations. **Results:** Conditional logistic FE regressions showed that the onset of a stroke or other forms of cerebrovascular disease was not associated with an increased likelihood of experiencing fear of falling (OR = 1.25, *p* = 0.095). However, stratified by sex, such an association was present in men (OR = 1.79, *p* = 0.006), though not in women (OR = 0.94, *p* = 0.732). **Conclusions:** The onset of a stroke or other cerebrovascular diseases was associated with an increased likelihood of experiencing FOF in men but not women. Efforts are required to assist older men in avoiding FOF after the onset of stroke or other cerebrovascular pathologies.

## 1. Introduction

A stroke, as the best-known type of cerebrovascular disease, is in the most general way defined as an acute or chronic damaging of central nervous tissue due to vascularly pathologies, with a multitude of additional possible clinical symptoms or imaging criteria [1,2]. As one of the leading causes of mortality and morbidity in older people [3,4], strokes are usually associated with long-term disability and can lead to reduced quality of life [4,5,6]. Stroke survivors consequently suffer from an increased likelihood of experiencing depression [7,8,9], loneliness [10], increased falls [11,12,13], or diminishing levels of participation in their communities and physical activities [12,14,15,16].

Included in the term cerebrovascular disease by most common clinic definitions are several different forms of pathologies in the extra- or intracranial vascular system (for example, stenosis, inflammation, dissection, etc.), usually increasing the likelihood of acute or chronic damaging of brain tissue.

The incidence of strokes is expected to remain high or even rise worldwide in the next years, primarily due to increases in age of the population and sedentary lifestyle in many countries [4,17,18]. This, in combination with an increased likelihood of surviving a stroke (at least in high-income countries) [19,20], also increases the prevalence of stroke survivors with all their post-stroke complications, including often permanent disabilities. This increases the need for further prevention and intervention in the interest of individual and public health [21,22]. Consequently, many medical interventions are trying to reduce the modifiable risk factors of strokes like hypertension, smoking, and sedentary lifestyle [23].

It is quite well established that fear of falling (FOF) reduces participation in the community and decreases physical activities [12,14,15,16,24,25,26] among older adults (particularly among stroke survivors). Moreover, FOF reduces participation in physical medical interventions [27] and increases the likelihood of falling [13] in such groups. In sum, these factors make it an important factor to consider and intervene in for successful ageing in older patients. Examining whether strokes are associated with developing of FOF in older adults could be a worthwhile addition towards improving care and targeted intervention for the already vulnerable population of stroke survivors.

Several studies have already examined the association between cerebrovascular disease, mainly stroke in that regard, and FOF in several ways, with three main groups emerging. The first are qualitative studies [25,28,29], the second are cross-sectional studies of local older adults or cohorts from medical facilities [30,31,32,33,34] and the third are longitudinal observations, usually without matched controls [35,36,37]. One large longitudinal cohort study from the USA did use matched controls comparing, among other things, the chance of developing FOF between people with and without strokes, but did not examine the association between the onset of stroke and FOF [13].

The previous literature describing the incidence and severity of strokes compared between the sexes suggested differences between men and women, although the results and directions of that difference are somewhat contradictory across the different papers [38,39,40]. As described above, with FOF being a well-established factor, differences in the prevalence between the sexes [13,41], and previous studies suggesting men to be less likely to seek medical help [42], exploring differences between the sexes in risk factor could prove to be a worthwhile effort in improving successful ageing and directing public health resources accordingly.

An association between stroke and FOF could emphasize the importance of further primary prevention of stroke and specific targeted interventions in rehabilitation of stroke survivors, improving quality of life and successful ageing. To the best of our knowledge, there has not been a single longitudinal analysis using panel data regressions with a large cohort investigating the potential association of the incidence of strokes or other cerebrovascular diseases and developing FOF (also stratified by sex). Thus, the aim of this study was to clarify the link between the onset of cerebrovascular diseases and developing FOF among middle-aged and older adults in Europe.

## 2. Materials and Methods

### 2.1. Sample

The data for this analysis came from the Survey of Health, Ageing and Retirement in Europe (SHARE) [43,44]. SHARE is a multinational survey of community-dwelling people aged 50 years or above, including their spouses. Since the 7th wave, SHARE includes all European Union member countries as well as Israel. Starting from 2004, the biennial survey includes now up to nine waves, with a mixture of both cross-sectional and longitudinal data. The response rate for each wave is about 40–50%, the individual retention rate in-between waves is described as around 70% [45]. Further details on the mixture and data collected by SHARE and how certain variables, like sex of the participants, were determined are provided elsewhere [43].

Data was collected with the help of computer-assisted personal interviews (CAPI). Prior consent was acquired verbally, as it was approved by the ethics commission. For this analysis, the longitudinal data from wave 5 (2013), wave 6 (2015), and wave 7 (2017) were used. Prior waves are excluded due to data availability and later waves due to the influence of the COVID-19 pandemic for data collection and the unprecedented restrictions of mobility and social interaction. The analytical sample (among the total sample) equalled *n* = 22,071 observations.

### 2.2. Dependent Variables

FOF was dichotomously assessed with the question “For the past six months at least, have you been bothered by any of the health conditions on this card?”, with four options available, one of them being “fear of falling down”. This dichotomous approach is common, particularly in larger cohort studies [13,30,37,46].

### 2.3. Independent Variables

To assess stroke, the participants were asked the question “Has a doctor ever told you that you had/Do you currently have”, with “stroke or cerebrovascular disease” as one of the options available to be selected (1. a heart attack; 2. high blood pressure or hypertension; 3. high blood cholesterol; 4. a stroke or cerebral vascular disease; 5. diabetes or high blood sugar; 6. chronic lung disease; 7. cancer or malignant tumour; 8. stomach or duodenal ulcer, peptic ulcer; 9. Parkinson’s disease; 10. cataracts; 11. hip fracture or femoral fracture). This self-reporting is a typical way of asking for health conditions in large (multinational) surveys with many aspects of life questioned [30,47], particularly longitudinal ones [13,47]. To improve readability, we will continue to use the term stroke referring to both strokes and cerebrovascular diseases for this work.

As potential time-varying confounding factors, we included in the analysis these sociodemographic factors: age, current employment (retired; employed or self-employed; unemployed; permanently sick or disabled; homemaker), marital status (married and living together with spouse; registered partnership; married, living separated from spouse; never married; divorced; widowed), and area of housing (a big city; the suburbs or outskirts of a big city; a large town; a small town; a rural area or village).

To consider the personal health and well-being of the participant, we included self-rated health status (single item from 1 to 5, excellent to poor) and quality of life (CASP-12: control, autonomy, pleasure and self-realization, score from 12 to 48, with higher scores indicating better quality of life) [48,49] as suggested by previous works [50], number of chronic diseases excluding stroke (0–10, as seen above), BMI category (underweight being < 18.5 kg/m^2^, normal weight between 18.5 kg/m^2^ and 24.9 kg/m^2^, overweight between 25 kg/m^2^ and 29.9 kg/m^2^, and obese 30 kg/m^2^ and above), and other health measures (in the last six months, did you experience any of these: fatigue; falls; dizziness/blackouts/faints). To assess cognition, we included results from the 10-word-recall [51] and 10-word delayed recall test [52] (0–10, with higher scores indicating better cognitive performances) [53]. To adjust for functional and motoric decline, we included the activities of daily living (0–5, sum score of reported difficulties with the following: dressing; bathing or showering; eating; cutting up food; walking across a room; getting in or out of bed) [54], instrumental activities of daily living (0–5, sum score of reported difficulties with the following: telephone calls; taking medications; managing money; shopping for groceries; and preparing a hot meal) [55] as used by previous works [56,57,58], as well as motor limitations with a sum of difficulties with ten different tasks ranging from basic fine (like picking up a small coin from a table) to gross (like walking 100 m) motor capabilities, as suggested by a previous study [59].

### 2.4. Statistical Analysis

A common source of bias in observational, cross-sectional studies is unobserved heterogeneity. Panel regression models can assist in mitigating the bias of unobserved heterogeneity [60]. For random effects analyses, one must assume no association between explanatory variables (like stroke, or FOF in our study) and time-invariant variables (like genetic predisposition). This, as for example with genetic differences, is a presumably unrealistic assumption to make. Linear fixed effects regression analysis can provide consistent estimates even when time-invariant factors (both observed and unobserved) are associated with the explanatory variables. Thus, we used conditional FE logistic regressions (also stratified by sex).

This model of panel regression data only includes the information of individuals who experienced a change within their FOF status over time. In this context, for example, the information of individuals who changed their status between wave 5 and wave 7 from not experienced stroke or cerebrovascular diseases to experienced stroke were included in the conditional FE regressions.

**Figure A1:**
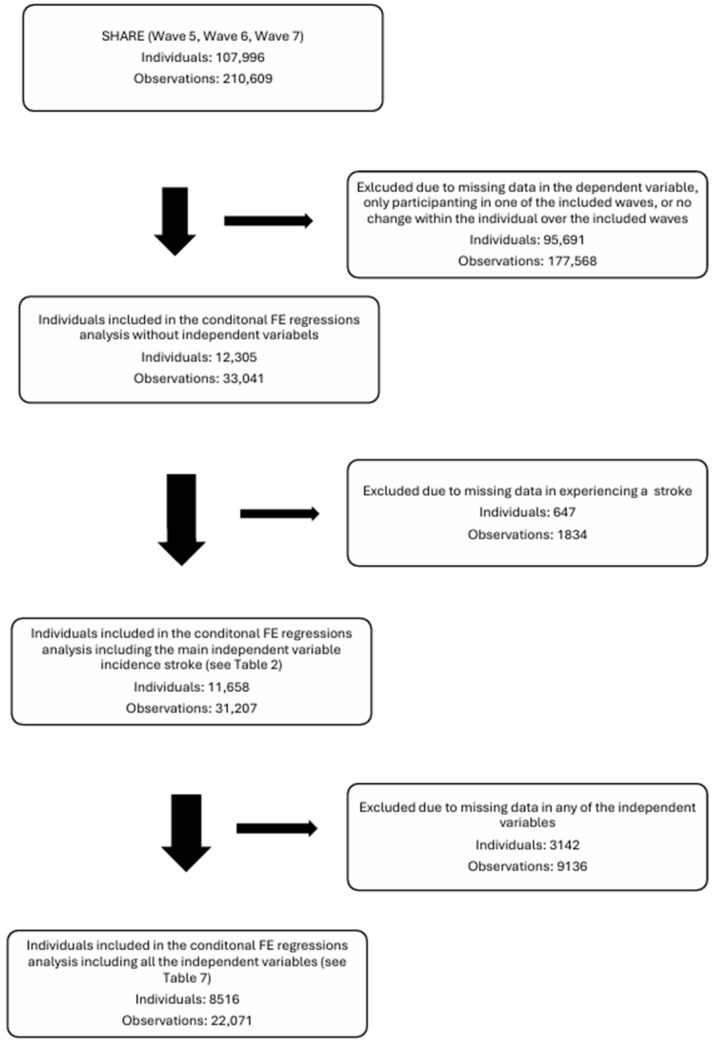
Flow chart of the included individuals and observations at each step of the conditional FE regressions analysis.

The change in an individual’s FOF status over the included waves was analysed and correlated with the reported change in all the included time-varying independent variables within the same individual, including experiencing stroke or other cerebrovascular diseases. Consequently, participants who participated in just one of the included waves questionnaires, or participants who did not experience a change in FOF during the observed period between wave 5 and 7, were excluded from the conditional FE logistic regression analysis since this analytical approach focuses on changes within individuals over time (see Figure A1 in the Appendix A). However, of note, this is not a shortcoming of a FE strategy. It rather reflects the fact that only a certain proportion of individuals in the population experience such a change over time.

We successively included the independent variables in the multivariate analysis in multiple steps. This resulted in a total of five different models, each further inclusion being described in the paragraph below: first sociodemographic factors, then adding well-being according to CASP-12, then personal health, and after that cognition, and finally functional/motoric abilities.

A *p* value of <0.05 was set as statistically significant. Stata 17.1 (StataCorp, College Station, TX, USA) was used for data analysis.

## 3. Results

### 3.1. Sample Characteristics of the General and Analysed Population

Table 1 shows characteristics of the included observations in the FE logistic regression stratified by sex. The average age was 72.6 years (SD 9.4 years) among men and 71.0 years (SD 9.7 years) among women. In total, 69.3% of the participants were female. It is worth noting that women, in comparison to men, were more often homemakers (13.8% compared to 0.3%) and considered themselves less often retired (68.7% compared to 82.6%) and more often widowed (26.6% compared to 10.3%). Among men, about 7.3% had experienced a stroke, whereas this value was 3.6% among women.

### 3.2. Unadjusted Regression Analysis

Table 2 shows the unadjusted fixed effects odds ratio for the link between stroke and FOF adjusted by sex. For all participants, experiencing a stroke increased the odds of FOF (OR = 2.32, 95% CI: 1.95–2.77, *p* < 0.001). Stratified by sex, male participants experienced a statistically significant increase in FOF after having a stroke (OR = 3.30, 95% CI: 2.51–4.33, *p* < 0.001). Moreover, female participants also experience a significant increase in FOF after having a stroke (OR = 1.75, 95% CI: 1.38–2.21, *p* < 0.001).

### 3.3. Adjusted Regression Analysis

Table 3 shows the results of the conditional FE logistic regression analysis including age and other sociodemographic factors. Male participants still showed a significant increase in developing FOF but not female participants. Successively including other time-varying covariates like well-being (Table 4), personal health (Table 5), and cognition (Table 6) still resulted in significantly increased odds of experiencing FOF in male participants after experiencing a stroke, but this did not hold in female participants. Table 7 shows the results of the fully adjusted conditional FE logistic regression analysis including sociodemographic, cognition, health, and well-being, as well as functional and motoric abilities. Having experienced a stroke only showed a borderline statistically insignificant association with the odds of experiencing FOF in the past 6 months (OR = 1.25, *p* = 0.095). Stratified by sex, the male participants showed a significant increase in the odds of developing FOF (OR: 1.79, 95% CI: 1.18–2.71), with female participants showing no significant association between stroke and FOF (OR: 0.94, 95% CI: 0.67–1.32)).

## 4. Discussion

### 4.1. Main Findings

The aim of this longitudinal study based on a large multinational sample from the SHARE study was to investigate the link between experiencing cerebrovascular diseases, mostly strokes, and developing FOF, adjusting for a variety of time-varying socio-demographic and health-related factors. Adjusted regressions showed that there is a link between the onset of a stroke and developing FOF among men but not in women. This longitudinal study adds to the existing knowledge, mainly based on cross-sectional studies regarding the association between stroke and FOF [13,59,61].

### 4.2. Relation to Previous Research

Several—mainly cross-sectional—studies already showed a cross-sectional association between stroke and higher odds of FOF [13,25,30,35]. More precisely, previous studies analysing this topic consisted either of smaller (hospital) cohorts (Goh et al., 2016; Schmid and Rittman, 2007; Schmid et al., 2011) or general observations on the development of FOF in subacute or chronic stroke victims without comparisons to people without stroke [34,36,37] or cross-sectional study designs [30,31,32,33]. In sum, all these studies included only patients with prevalent stroke (as opposed to incident stroke) or only the development of FOF in cohorts exclusively among stroke victims. There is a complete lack of studies investigating whether the incidence of stroke is associated with changes in the likelihood of developing FOF. Thus, our current work extends previous work by showing that the onset of stroke is associated with an increased likelihood of FOF among older men longitudinally.

Why might a stroke contribute to the development of a fear of falling? As previously described, strokes can lead to serious and permanent loss of cognitive as well as motor functioning. Qualitative work in this field suggests that the realization of sudden or slow limitations in one’s bodily functions, and especially the loss of control over these circumstances, is one of the main factors contributing to the increase in FOF in older age [62]. A previous work using different waves from SHARE found an influence of the sum of motor limitations on the development of FOF [59], as well as interviews from stroke survivors suggesting that changes in motor functioning are one of the main causes of developing FOF after stroke [29]. However, our study was successively adjusted for motor and essential activities of daily living limitations. In tendency, the OR did decline after including these variables like dizziness or declining motor functions, which could and are influenced by experiencing a stroke. This decrease in the OR was more pronounced with female participants, who, after including these variables, did not show any significant increase in OR in experiencing FOF after stroke. These results strongly suggest that part of the increase in the OR of FOF after stroke is mediated through these parameters. But for male participants, it remained still strongly significant. SHARE does not provide distinctions on severity on some of these parameters, like falls experienced or the level of dizziness. Additionally, SHARE did not provide further parameters which could possibly have mediated the effects of stroke on FOF (like, for example, changes in gait). Further research including validated parameters to measure the amount of physical and mental changes in the context of the incident of stroke are needed to further distinguish between the mediating parameters and the reason for differences in this mediation between the sexes.

Furthermore, experiencing a stroke often leads to sudden relatively long hospital and rehabilitation stays. This loss of autonomy, even if it is only temporary, could lead to a long-lasting decrease in trust of one’s abilities and increases of fears related to that. Future research is required to test this potential pathway.

Our analysis showed a significant longitudinal association between the incidence of stroke and developing FOF in men but not in women. This result is in line with a cross-sectional survey of community-dwelling older adults in Taiwan [30]. However, our current findings are in contrast to results from a cross-sectional work on sub-acute stroke victims [34] and a large longitudinal study finding that stroke was positively associated with FOF, particularly among women [13]. These differences can most likely be explained with differences in design and analytical approaches. The hospital cohort of sub-acute stroke victims [34] did not have any healthy matched controls, and the longitudinal study [13] only questioned history of comorbidities as strokes at the baseline home interview, compared to our longitudinal study examining the association between the onset of stroke and FOF.

Are stroke severity differences between sexes the main reason for this discrepancy? The literature on the differences between the severity and residual effects of strokes in male or female patients, as well as length of hospital stays, is somewhat contradictory and varies by country. Some studies observed a higher mortality and overall residual disability rate and hospital stays with female stroke victims [38]. Others observed higher residual disability but shorter hospital stays [39], no differences in residual disability or hospital stay [40], or female patients having a better functional outcome and recovery compared to male patients [63,64]. Thus, with a lack of longitudinal data and overall inconclusive results, future research is required to evaluate the influence of potential sex differences in post-stroke severity and residuality of FOF.

Another possible explanation for these differences could be different attitudes to medical interventions as well as medical professionals. The few existing works suggest that men in general seek less medical help than women [42], and the generally higher reporting of FOF from women [13,41] could lead to medical professionals underestimating the need for help among men in providing interventions and walking aid. However, to the best of our knowledge, there is a lack of studies examining these factors. Thus, future research is needed to test this in further detail.

Other explanation approaches may refer to differences in reporting FOF between women and men prior to experiencing stroke, with women more often reporting FOF before strokes and men increasing afterwards. This could explain the lack of increase in FOF in female patients after stroke since most of the vulnerable population already reports it before. Future research could focus on changes in intensity of FOF to further differentiate these subtleties. Another factor might be differences in coping styles, but little to no research exists dealing with specific differences in coping strategies between different demographic groups like sex [65,66]. Future research is required to further analyse these associations.

Furthermore, recent qualitative approaches have questioned the conception of FOF as being purely negative factor, especially in falls efficacy, and suggested other mechanism as mediators for some of the negative influences, like, for example, anxiety [67]. Future surveys and research with more subtle analysis of the emotional state of the participants would be needed to provide more detailed methods of describing mediating factors between FOF and stroke and the adverse effects, as described in the above paragraphs.

Experiencing a stroke will continue to constitute a critical event in an individual’s life and future well-being, especially in vulnerable populations with potentially few resources to compensate for them like older patients. Short- and long-term rehabilitation remains a challenging balance act of patients’ needs and limited resources in everyday clinical work and the attempt to prevent chronic immobilization. Identifying potential mediators of immobilization, like FOF, and identifying vulnerable sub-populations and circumstances of manifestation, such as the above-mentioned anxiety level of a patient, could prove useful tools for allocating resources in everyday clinical and rehabilitative care and improving long-term successful ageing.

### 4.3. Strengths and Limitations

The use of SHARE data (a large representative pan-European survey) is a strength of our study. Moreover, various covariates were included in our study. Furthermore, longitudinal data (three waves) were used. This allowed us to combine the positive aspects of previous research dominating in this field, such as large cross-sectional studies [30] and the longitudinal data of smaller hospital studies [36]. Additionally, the use of FE regressions mitigates the challenge of unobserved heterogeneity [60].

The main dependent variable FOF was, as was mentioned in the methods part, dichotomously questioned with a Yes or No answer, which has been shown to underestimate prevalence of FOF in questioned populations [68]. This, with its high face validity, is, however, a common approach in studies specifically analysing the concept of FOF [13,30,34,37,46], as other scales commonly used in studies, like FES-1 or ABC, technically examine other concepts related but not identical to FOF [68]. Other tools to examine FOF in more detail do exist, like the SAFE scale or modifications of it, but are generally described as too laborious to be widely adopted [68], even more so in such a large and continuous survey as SHARE, which focuses on many different aspects of the life of its participants [43].

Similarly, experiencing a stroke was questioned through self-reporting of the participants, and again in terms of yes or no, explicitly including other forms of cerebrovascular disease not further specified by the data. Furthermore, in the standardized questionnaire, SHARE only provided stroke as an example of cerebrovascular disease. Many older participants may struggle with further understanding the subtleties and differences between the different pathologies included in this category, leading to possible underreporting in this category. It would be preferable to extract such information through more objective means like clinical records, which could also provide further details with type and initial severity of stroke [24,34,37,69] or other forms of included cerebrovascular diseases as defined by professionals (with only limited knowledge to be expected in the nuances of different cerebrovascular pathologies by non-medical professional participants in the survey). This, however, would naturally limit the amount of people reached, as well as exclude people in general who do not regularly participate in the healthcare system. Consequently, many studies use this approach of self-reported personal health history, especially with large surveys or cohorts [13,30,47]. Furthermore, only a few studies with small hospital cohort sizes have been able to consider the type and more specifically the severity of the initial stroke [24,34,37]. Additionally, a small sample selection and a small attrition bias have been identified in the SHARE study [70].

## 5. Conclusions

This study showed a longitudinal association between stroke and FOF among men. Efforts are required to assist older men in avoiding FOF after stroke. This could contribute to successful ageing in later life. Future research based on longitudinal studies from other regions is required to confirm our current findings.

## Figures and Tables

**Table 1:** Characteristics of the analysed population in the adjusted FE regression analysis.

Variables	Sex	
	Male	Female
Sex	N = 6786	N = 15,285
	N (%)/Mean (SD)	N (%)/Mean (SD)
--sociodemographic factors--		
Age in years	72.6 (9.4)	71.0 (9.7)
Relationship status		
Married and living together with spouse	4939 (72.8%)	8556 (56.0%)
Registered partnership	72 (1.1%)	102 (0.7%)
Married, living separated from spouse	71 (1.0%)	164 (1.1%)
Never married	469 (6.9%)	761 (5.0%)
Divorced	535 (7.9%)	1629 (10.7%)
Widowed	700 (10.3%)	4073 (26.6%)
Area of living		
A big city	917 (13.5%)	2370 (15.5%)
The suburbs or outskirts of a big city	585 (8.6%)	1341 (8.8%)
A large town	988 (14.6%)	2497 (16.3%)
A small town	1890 (27.9%)	4091 (26.8%)
A rural area or village	2406 (35.5%)	4986 (32.6%)
Employment situation		
Retired	5607 (82.6%)	10,497 (68.7%)
Employed or self-employed (including working for family)	649 (9.6%)	1664 (10.9%)
Unemployed	141 (2.1%)	262 (1.7%)
Permanently sick or disabled	371 (5.5%)	749 (4.9%)
Homemaker	18 (0.3%)	2113 (13.8%)
--personal health and well-being--		
Experienced a stroke during observation		
No	6294 (92.7%)	14,742 (96.4%)
Yes	492 (7.3%)	543 (3.6%)
Experienced FOF during the last 6 months		
No	3789 (55.8%)	8247 (54.0%)
Yes	2997 (44.2%)	7038 (46.0%)
Experienced a fall during the last 6 months		
No	5795 (85.4%)	12,739 (83.3%)
Yes	991 (14.6%)	2546 (16.7%)
Experienced fatigue during the last 6 months		
No	4590 (67.6%)	10,053 (65.8%)
Yes	2196 (32.4%)	5232 (34.2%)
Experienced faints, dizziness, or blackouts during at least the last 6 months		
No	4996 (73.6%)	10,833 (70.9%)
Yes	1790 (26.4%)	4452 (29.1%)
Self-rated health (1–5, the lower the number the better the self-rated health)	3.7 (0.9)	3.6 (0.9)
Number of chronic diseases	1.7 (1.4)	1.5 (1.3)
CASP-12 quality of life score (12–48, the higher the score, the better the quality of life)	35.0 (6.2)	35.3 (6.3)
BMI		
Below 18.5—underweight	49 (0.7%)	265 (1.7%)
18.5–24.9—normal	1949 (28.7%)	4716 (30.9%)
25–29.9—overweight	2897 (42.7%)	5686 (37.2%)
30 and above—obese	1891 (27.9%)	4618 (30.2%)
--functional and motoric abilities--		
Instrumental activities of daily living indices (0–5, the higher the number, the greater the difficulties with the activities)	0.3 (0.8)	0.2 (0.7)
Activities of daily living index (0–5, the higher the number, the greater the difficulties with the activities)	0.4 (0.9)	0.3 (0.8)
Mobility impairment (0–10, the higher the number, the greater the difficulties with mobility)	2.7 (2.6)	3.0 (2.5)
--cognition--		
10 word recall test (0–10, the higher the number, the better the cognition)	4.6 (1.7)	5.1 (1.8)
10 word delayed recall test (0–10, the higher the number, the better the cognition)	3.1 (2.0)	3.7 (2.2)

**Table 2:** Developing FOF in the last 6 months after experiencing a stroke: unadjusted conditional FE logistic regressions (also stratified by sex).

	All Participants	Male	Female
Incident stroke	2.32 *** (1.95–2.77)	3.30 *** (2.51–4.33)	1.75 *** (1.38–2.21)
Observations	31,207	9651	21,556
Number of Individuals	11,658	3640	8018
Pseudo R^2^	0.00411	0.0119	0.00143

Notes: unadjusted odds ratio; 95% CI in parentheses, *** *p* < 0.001.

**Table 3:** Developing FOF in the last 6 months after experiencing a stroke: adjusted conditional FE logistic regressions (also stratified by sex) including sociodemographic factors.

Variables	All Participants	Male	Female
Incident stroke	1.72 ***	2.30 ***	1.27 +
	(1.40–2.10)	(1.69–3.12)	(0.97–1.67)
Time-varying covariates:			
Sociodemographic factors			
Age in years	1.15 ***	1.19 ***	1.13 ***
	(1.13–1.16)	(1.16–1.22)	(1.11–1.15)
Relationship status (reference category: “Married and living together”)			
Registered partnership	0.82	1.17	0.68
	(0.28–2.35)	(0.16–8.37)	(0.19–2.40)
Married, living separated from spouse	1.09	0.45	1.40
	(0.43–2.76)	(0.04–4.89)	(0.50–3.90)
Never married	0.39	0.68	0.15 +
	(0.11–1.33)	(0.14–3.25)	(0.02–1.42)
Divorced	1.06	1.19	1.02
	(0.56–1.99)	(0.40–3.56)	(0.47–2.23)
Widowed	1.05	1.14	1.05
	(0.87–1.28)	(0.75–1.73)	(0.84–1.31)
Employment status (reference category: “Retired”)			
Employed or self-employed (including working for family business)	0.85 +	0.78	0.90
	(0.72–1.01)	(0.58–1.05)	(0.73–1.11)
Unemployed	1.21	1.20	1.24
	(0.93–1.57)	(0.76–1.91)	(0.90–1.71)
Permanently sick or disabled	1.37 ***	1.58 **	1.28 *
	(1.16–1.62)	(1.20–2.09)	(1.04–1.57)
Homemaker	1.01	1.97	0.98
	(0.87–1.18)	(0.73–5.30)	(0.84–1.15)
Area of living (reference category: “Village”)			
A big city	0.99	1.50 *	0.83
	(0.82–1.19)	(1.06–2.13)	(0.67–1.04)
The suburbs or outskirts of a big city	0.91	1.02	0.86
	(0.77–1.07)	(0.76–1.38)	(0.71–1.05)
A large town	0.99	1.27 +	0.89
	(0.85–1.15)	(0.97–1.67)	(0.74–1.06)
A small town	1.08	1.29 *	1.00
	(0.96–1.21)	(1.05–1.58)	(0.87–1.14)
Observations	26,958	8510	18,448
Number of individuals	10,220	3251	6969
Pseudo R^2^	0.0287	0.0533	0.0205

Notes: odds ratios are displayed; 95% CI in parentheses; *** *p* < 0.001, ** *p* < 0.01, * *p* < 0.05, + *p* < 0.10.

**Table 4:** Developing FOF in the last 6 months after experiencing a stroke: adjusted conditional FE logistic regressions (also stratified by sex) including sociodemographic factors as well as well-being according to the CASP-12 quality of life score.

Variables	All Participants	Male	Female
Incident stroke	1.76 ***	2.45 ***	1.31 +
	(1.41–2.21)	(1.71–3.52)	(0.98–1.76)
Time-varying covariates:			
Sociodemographic factors			
Age in years	1.14 ***	1.18 ***	1.12 ***
	(1.12–1.15)	(1.15–1.22)	(1.10–1.14)
Relationship status (Reference category: “Married and living together”)			
Registered partnership	0.97	1.28	0.80
	(0.32–2.90)	(0.17–9.62)	(0.20–3.17)
Married, living separated from spouse	1.19	0.55	1.57
	(0.44–3.18)	(0.05–6.06)	(0.52–4.76)
Never married	0.37	0.80	0.00
	(0.09–1.42)	(0.17–3.81)	(0.00–>>1000 ^§^)
Divorced	1.17	1.17	1.22
	(0.61–2.24)	(0.39–3.53)	(0.55–2.70)
Widowed	1.04	1.12	1.03
	(0.84–1.28)	(0.70–1.78)	(0.81–1.32)
Employment status (Reference category: “Retired”)			
Employed or self-employed (including working for family business)	0.86	0.85	0.87
	(0.72–1.03)	(0.62–1.17)	(0.70–1.09)
Unemployed	1.15	1.22	1.15
	(0.87–1.51)	(0.75–1.99)	(0.83–1.60)
Permanently sick or disabled	1.23 *	1.37 *	1.18
	(1.02–1.48)	(1.01–1.88)	(0.94–1.48)
Homemaker	1.00	2.36	0.96
	(0.85–1.17)	(0.80–7.02)	(0.81–1.13)
Area of living (Reference category: “Village”)			
A big city	1.00	1.78 **	0.80 +
	(0.82–1.22)	(1.21–2.61)	(0.63–1.02)
The suburbs or outskirts of a big city	0.90	1.07	0.85
	(0.76–1.08)	(0.77–1.48)	(0.69–1.04)
A large town	0.97	1.29 +	0.87
	(0.83–1.14)	(0.96–1.74)	(0.72–1.05)
A small town	1.07	1.37 **	0.97
	(0.95–1.21)	(1.10–1.71)	(0.84–1.12)
Well being			
CASP-12 quality of life score (12–48, the higher the score, the better the quality of life)	0.95 ***	0.95 ***	0.95 ***
	(0.94–0.96)	(0.94–0.96)	(0.94–0.96)
Observations	23,834	7286	16,548
Number of individuals	9141	2821	6320
Pseudo R^2^	0.0408	0.0703	0.0318

Notes: odds ratios are displayed; 95% CI in parentheses; *** *p* < 0.001, ** *p* < 0.01, * *p* < 0.05, + *p* < 0.10; § denotes number being >>1000.

**Table 5:** Developing FOF in the last 6 months after experiencing a stroke: adjusted conditional FE logistic regressions (also stratified by sex) including sociodemographic factors as well as personal health and well-being.

Variables	All Participants	Male	Female
Incident stroke	1.44 **	2.07 ***	1.07
	(1.12–1.85)	(1.39–3.09)	(0.77–1.49)
Time-varying covariates:			
Sociodemographic factors			
Age in years	1.11 ***	1.14 ***	1.10 ***
	(1.10–1.13)	(1.11–1.18)	(1.08–1.12)
Relationship status (Reference category: “Married and living together”)			
Registered partnership	0.89	1.12	0.69
	(0.28–2.79)	(0.11–11.46)	(0.17–2.82)
Married, living separated from spouse	1.14	0.30	1.57
	(0.40–3.23)	(0.02–4.94)	(0.49–5.02)
Never married	0.38	1.15	0.00
	(0.09–1.63)	(0.22–6.13)	(0.00–>>1000 ^§^)
Divorced	1.33	1.61	1.27
	(0.64–2.73)	(0.51–5.15)	(0.50–3.23)
Widowed	1.01	1.19	0.97
	(0.80–1.28)	(0.71–1.99)	(0.74–1.27)
Employment status (Reference category: “Retired”)			
Employed or self-employed (including working for family business)	0.88	0.91	0.87
	(0.73–1.07)	(0.65–1.27)	(0.69–1.10)
Unemployed	1.04	1.11	1.05
	(0.77–1.40)	(0.66–1.88)	(0.73–1.50)
	(0.88–1.31)	(0.87–1.71)	(0.80–1.31)
Homemaker	0.97	2.20	0.93
	(0.81–1.16)	(0.70–6.90)	(0.77–1.12)
Area of living (Reference category: “Village”)			
A big city	0.99	1.71 *	0.81
	(0.80–1.23)	(1.12–2.59)	(0.63–1.05)
The suburbs or outskirts of a big city	0.91	1.06	0.86
	(0.76–1.10)	(0.74–1.50)	(0.69–1.08)
A large town	0.98	1.27	0.88
	(0.82–1.16)	(0.92–1.74)	(0.72–1.08)
A small town	1.06	1.33 *	0.97
	(0.93–1.21)	(1.05–1.68)	(0.83–1.13)
Personal health and well-being			
Experienced a fall during the last 6 months (Ref.: No)	2.43 ***	2.65 ***	2.35 ***
	(2.23–2.66)	(2.23–3.14)	(2.12–2.61)
Experienced fatigue during the last 6 months (Ref.: No)	1.30 ***	1.26 ***	1.31 ***
	(1.20–1.40)	(1.10–1.45)	(1.19–1.43)
Experienced faints, dizziness, or blackouts during at least the last 6 months (Ref.: No)	1.86 ***	2.03 ***	1.80 ***
	(1.72–2.02)	(1.75–2.36)	(1.64–1.98)
Self-rated health (1–5, the lower the number, the better the self-rated health)	1.37 ***	1.41 ***	1.35 ***
	(1.30–1.44)	(1.29–1.54)	(1.27–1.44)
Number of chronic diseases (0–9)	1.11 ***	1.11 ***	1.11 ***
	(1.07–1.15)	(1.05–1.18)	(1.07–1.16)
CASP-12 quality of life score (12–48, the higher the score, the better the quality of life)	0.97 ***	0.98 **	0.97 ***
	(0.97–0.98)	(0.96–0.99)	(0.96–0.98)
BMI (Reference category: “18.5 kg/m^2^–24.9 kg/m^2^—normal”)			
Below 18.5 kg/m^2^—underweight	1.18	0.74	1.29
	(0.83–1.67)	(0.33–1.66)	(0.88–1.89)
25 kg/m^2^–29.9 kg/m^2^—overweight	0.97	0.94	0.99
	(0.87–1.10)	(0.77–1.16)	(0.86–1.14)
30 kg/m^2^ and above—obese	0.98	0.83	1.06
	(0.84–1.16)	(0.61–1.12)	(0.87–1.29)
Observations	22,687	7030	15,657
Number of individuals	8734	2726	6008
Pseudo R^2^	0.112	0.149	0.0990

Notes: odds ratios are displayed; 95% CI in parentheses; *** *p* < 0.001, ** *p* < 0.01, * *p* < 0.05; § denotes number being >>1000.

**Table 6:** Developing FOF in the last 6 months after experiencing a stroke: adjusted conditional FE logistic regressions (also stratified by sex) including sociodemographic factors as well as personal health and well-being and cognition.

Variables	All Participants	Male	Female
Incident stroke	1.38 *	1.98 ***	1.03
	(1.07–1.78)	(1.32–2.96)	(0.74–1.44)
Time-varying covariates:			
Sociodemographic factors			
Age in years	1.12 ***	1.15 ***	1.10 ***
	(1.10–1.13)	(1.11–1.18)	(1.08–1.12)
Relationship status (Reference category: “Married and living together”)			
Registered partnership	0.89	1.14	0.69
	(0.28–2.81)	(0.11–11.52)	(0.17–2.81)
Married, living separated from spouse	1.15	0.31	1.56
	(0.40–3.26)	(0.02–4.90)	(0.49–4.97)
Never married	0.37	1.16	0.00
	(0.09–1.63)	(0.22–6.08)	(0.00–>>1000 ^§^)
Divorced	1.33	1.75	1.22
	(0.64–2.74)	(0.53–5.78)	(0.49–3.07)
Widowed	1.02	1.18	0.98
	(0.80–1.29)	(0.70–1.97)	(0.75–1.28)
Employment status (Reference category: “Retired”)			
Employed or self-employed (including working for family business)	0.89	0.92	0.88
	(0.74–1.08)	(0.66–1.29)	(0.70–1.12)
Unemployed	1.03	1.09	1.05
	(0.77–1.39)	(0.64–1.85)	(0.73–1.51)
Permanently sick or disabled	1.06	1.19	1.02
	(0.87–1.30)	(0.85–1.67)	(0.79–1.31)
Homemaker	0.95	2.15	0.91
	(0.79–1.14)	(0.69–6.72)	(0.76–1.10)
Area of living (Reference category: “Village”)			
A big city	0.98	1.65 *	0.81
	(0.79–1.22)	(1.08–2.51)	(0.63–1.05)
The suburbs or outskirts of a big city	0.92	1.04	0.87
	(0.76–1.11)	(0.73–1.49)	(0.70–1.10)
A large town	0.98	1.25	0.89
	(0.82–1.17)	(0.91–1.73)	(0.72–1.10)
A small town	1.07	1.34 *	0.97
	(0.94–1.21)	(1.05–1.70)	(0.83–1.13)
Personal health and well-being			
Experienced a fall during the last 6 months (Ref.: No)	2.44 ***	2.61 ***	2.38 ***
	(2.23–2.67)	(2.19–3.10)	(2.14–2.64)
Experienced fatigue during the last 6 months (Ref.: No)	1.29 ***	1.26 **	1.30 ***
	(1.20–1.39)	(1.10–1.45)	(1.18–1.42)
Experienced faints, dizziness, or blackouts during at least the last 6 months (Ref.: No)	1.83 ***	2.00 ***	1.77 ***
	(1.69–1.99)	(1.72–2.33)	(1.61–1.95)
Self-rated health (1–5, the lower the number, the better the self-rated health)	1.37 ***	1.40 ***	1.35 ***
	(1.30–1.44)	(1.28–1.54)	(1.27–1.44)
Number of chronic diseases (0–9)	1.12 ***	1.12 ***	1.11 ***
	(1.08–1.16)	(1.05–1.19)	(1.07–1.16)
CASP-12 quality of life score (12–48, the higher the score, the better the quality of life)	0.97 ***	0.98 **	0.97 ***
	(0.97–0.98)	(0.96–0.99)	(0.96–0.98)
BMI (Reference category: “18.5 kg/m^2^–24.9 kg/m^2^—normal”)			
Below 18.5 kg/m^2^—underweight	1.22	0.77	1.32
	(0.85–1.75)	(0.33–1.82)	(0.89–1.97)
25 kg/m^2^–29.9 kg/m^2^—overweight	0.98	0.95	1.00
	(0.87–1.10)	(0.77–1.17)	(0.86–1.15)
30 kg/m^2^ and above—obese	0.99	0.84	1.07
	(0.84–1.17)	(0.62–1.14)	(0.88–1.30)
Cognition			
Ten word recall test (0–10, the higher the number, the better the cognition)	1.02	1.01	1.02
	(0.99–1.05)	(0.96–1.07)	(0.99–1.05)
Ten word delayed recall test (0–10, the higher the number, the better the cognition)	0.98	1.01	0.97 *
	(0.96–1.01)	(0.97–1.06)	(0.95–1.00)
Observations	22,241	6848	15,393
Number of individuals	8570	2659	5911
Pseudo R^2^	0.111	0.147	0.0988

Odds ratios are displayed; 95% CI in parentheses; *** *p* < 0.001, ** *p* < 0.01, * *p* < 0.05; § denotes number being >>1000.

**Table 7:** Developing FOF in the last 6 months after experiencing a stroke: adjusted conditional FE logistic regressions (also stratified by sex) including sociodemographic factors as well as personal health and well-being, cognition, and functional/motoric abilities.

Variables	All Participants	Male	Female
Incident stroke	1.25 +	1.79 **	0.94
	(0.96–1.62)	(1.18–2.71)	(0.67–1.32)
Time-varying covariates:			
Sociodemographic factors			
Age in years	1.10 ***	1.12 ***	1.09 ***
	(1.08–1.12)	(1.09–1.16)	(1.07–1.11)
Relationship status (Reference category: “Married and living together”)			
Registered partnership	0.86	1.32	0.62
	(0.26–2.86)	(0.11–15.76)	(0.14–2.67)
Married, living separated from spouse	1.03	0.31	1.32
	(0.35–2.99)	(0.02–5.79)	(0.41–4.27)
Never married	0.40	1.35	0.00
	(0.09–1.78)	(0.23–8.04)	(0.00–>>1000 ^§^)
Divorced	1.27	1.67	1.13
	(0.61–2.65)	(0.50–5.62)	(0.45–2.88)
Widowed	1.01	1.15	0.98
	(0.79–1.29)	(0.66–1.97)	(0.74–1.29)
Employment status (Reference category: “Retired”)			
Employed or self-employed (including working for family business)	0.88	0.88	0.89
	(0.72–1.08)	(0.62–1.24)	(0.70–1.13)
Unemployed	1.00	1.04	1.02
	(0.73–1.36)	(0.60–1.81)	(0.70–1.48)
Permanently sick or disabled	0.92	1.10	0.86
	(0.75–1.14)	(0.77–1.57)	(0.66–1.12)
Homemaker	1.02	2.59	0.97
	(0.85–1.23)	(0.80–8.35)	(0.80–1.18)
Area of living (Reference category: “Village”)			
A big city	0.98	1.64 *	0.81
	(0.78–1.22)	(1.06–2.54)	(0.63–1.06)
The suburbs or outskirts of a big city	0.89	0.93	0.88
	(0.74–1.09)	(0.64–1.34)	(0.70–1.11)
A large town	1.00	1.23	0.92
	(0.83–1.19)	(0.88–1.72)	(0.74–1.13)
A small town	1.07	1.32 *	0.98
	(0.94–1.23)	(1.03–1.69)	(0.84–1.15)
Personal health and well-being			
Experienced a fall during the last 6 months (Ref.: No)	2.32 ***	2.42 ***	2.28 ***
	(2.11–2.54)	(2.02–2.90)	(2.05–2.54)
Experienced fatigue during the last 6 months (Ref.: No)	1.18 ***	1.11	1.20 ***
	(1.09–1.27)	(0.96–1.28)	(1.09–1.32)
Experienced faints, dizziness, or blackouts during at least the last 6 months (Ref.: No)	1.72 ***	1.87 ***	1.67 ***
	(1.58–1.87)	(1.60–2.19)	(1.51–1.84)
Self-rated health (1–5, the lower the number, the better the self-rated health)	1.26 ***	1.26 ***	1.25 ***
	(1.19–1.32)	(1.15–1.39)	(1.17–1.33)
CASP-12 quality of life score (12–48, the higher the score, the better the quality of life)	0.99 ***	0.99	0.98 **
	(0.98–0.99)	(0.97–1.00)	(0.97–0.99)
Number of chronic diseases (0–9)	1.08 ***	1.08 *	1.08 ***
	(1.04–1.12)	(1.01–1.15)	(1.04–1.13)
BMI (Reference category: “18.5 kg/m^2^–24.9 kg/m^2^—normal”)			
Below 18.5 kg/m^2^—underweight	1.20	0.81	1.27
	(0.82–1.74)	(0.32–2.00)	(0.85–1.92)
25 kg/m^2^–29.9 kg/m^2^—overweight	0.98	0.93	1.01
	(0.87–1.11)	(0.75–1.15)	(0.87–1.17)
30 kg/m^2^ and above—obese	0.96	0.84	1.02
	(0.81–1.14)	(0.61–1.15)	(0.83–1.25)
Functional and motoric abilities			
Activities of daily living index (0–5, the higher the number, the greater the difficulties with the activities)	1.07 *	1.05	1.07 +
	(1.01–1.13)	(0.95–1.16)	(1.00–1.15)
Instrumental activities of daily living indices (0–5, the higher the number, the greater the difficulties with the activities)	1.04	1.04	1.05
	(0.98–1.12)	(0.93–1.16)	(0.96–1.14)
Mobility impairment (0–10, the higher the number, the greater the difficulties with mobility)	1.22 ***	1.25 ***	1.20 ***
	(1.19–1.24)	(1.21–1.31)	(1.17–1.23)
Cognition			
Ten word recall test (0–10, the higher the number, the better the cognition)	1.02	1.01	1.03
	(0.99–1.05)	(0.96–1.07)	(0.99–1.06)
Ten word delayed recall test (0–10, the higher the number, the better the cognition)	0.99	1.02	0.97 +
	(0.96–1.01)	(0.97–1.07)	(0.95–1.00)
Observations	22,071	6786	15,285
Number of individuals	8516	2639	5877
Pseudo R^2^	0.139	0.181	0.124

Notes: odds ratios are displayed; 95% CI in parentheses; *** *p* < 0.001, ** *p* < 0.01, * *p* < 0.05, + *p* < 0.10; § denotes number being >>1000.

## Data Availability

Restrictions apply to the availability of these data. Data were obtained from the SHARE Research Data Center (FDZ-SHARE) and are available after registration under/at https://share-eric.eu/data/data-access with the permission of SHARE-ERIC (Survey of Health, Ageing and Retirement in Europe—European Research Infrastructure Consortium).
